# 

**Published:** 1881-01

**Authors:** 


### INDEX

TO THE

### INDEPENDENT PRACTITIONER.

### VOLUME II.

PAGE.

A Simple Explanation........................................................ 96
A Simple Fire Escape........................................................340
A New Dental Disease........................................................695
Abortion With Retained Placenta........................................................731
About Coin........................................................137
Aconite in Remittent Fever........................................................704
Adam a Nation........................................................171
Adulterated Coffee........................................................ 289
After Thirty-eight Years Practice........................................................693
Air and Ventilation........................................................270, 352
Alcoholism a Cause of Crime and Epilepsy........................................................578
Alveolar Abscess........................................................199
Amalgam Plug, Death From........................................................199
American Association for the Advancement of Science........................................................654
American Dental Association, 21st Annual Meeting........................................................481
American Investigations in Turkey........................................................576
Amyl Nitrite as a Cardiac Stimulant........................................................698
Anæmia, Treatment of........................................................711
Anatomical and Physiological Differences Between the Caucasian and African Races........................................................348
Anesthetic Action of Chlorine........................................................424
Anesthetics and Narcotics........................................................161
Antipyretic Remedies........................................................700
Antiquities in New Mexico........................................................359
Antiseptics and Disinfectants........................................................367

Page.
Apocynum Cannabinum in Dropsy....................................119
Apocynum Cannabinum in Bright's Disease..........................306
Apomorphia in Diseases of Children...............................713
Apostate's Creed................................................. 49
Are All the Races of Men Descended from a Single Pair?..........274
Arctic Research..................................................214
Arnold, A. B., Notes on Some cases of Malarial Disease...........666
Arsenical Poisoning..............................................141
Artificial Impregnation..........................................712
Asthma...........................................................274
Atresia of Vagina and Uterus, Palliative Treatment of............751
Bad effects of Train Catching....................................291
Baldness, New Remedy For.........................................442
Barrett, W. C., Pus Dyscrasia.................................... 23
Bathing After Meals..............................................293
Beloved Physicians...............................................310
Benefits of Sanitation...........................................648
Benzoate of Calcium..............................................558
Beri-Beri........................................................147
Bernhardt Craze, The.............................................499

### *Bibliographical Department.*

Bartholow, R., Materia Medica and Therapeutics................... 39
Bartholow, R., Medical Electricity...............................257
Bartholow, R., Treatise on the Practice of Medicine.............. 40
Benedikt, M., Anatomical Studies upon Brains of Criminals........598
Day, Wm. H., The Diseases of Children............................255
Denison, C., Rocky Mountain Health Resorts....................... 86
Dental Directory of the World.................................... 42
Detroit Free Press............................................... 41
Directory of the Proceedings of the International Medical Congress.538
Duhring, L. A., A Practical Treatise on Diseases of the Skin......186
Edwards, Joseph L., How a Person Affected with Bright's Disease Ought to Live.....256
Garrettson, James E., A System of Oral Surgery...................322
Green, T. Henry, An Introduction to Pathology and Morbid Anatomy.....255
Gross, S. W., A Practical Treatise on Impotence, Sterility, &c....486

Page

Hale, Amie M., The Management of Children.........................256
Holden, L., Landmarks—Medical and Surgical.........................599
Index-Catalogue of Library of Surgeon-General's Office, Vol. 2...486
Medical Diagnosis, with Special Reference to Practical Medicine..124
Mitchell, S. Weir., Lectures on Diseases of the Nervous System, Especially in Women.........................254
New Exchanges.........................762
Packard, J. H., Sea-Air and Sea-Bathing.........................256
Popular Science Monthly.........................675
Smythe, G. C., Medical Heresies Historically Considered.......... 85
The Century Magazine.........................761
The Compend of Anatomy.........................599
The Microscope and its Application to Medicine and Pharmacy...539
Transactions of the American Medical Association.........................125
Transactions of the Eleventh Annual Session of the Medical Society of Virginia.........................125
Transactions of the Mississippi State Medical Association...........125
Thomas, T. Gaillard, A Practical Treatise on Diseases of Women. 84
Turnbull, L., Imperfect Hearing and the Hygiene of the Ear......187
VanBuren, W. H., Lectures upon Diseases of the Rectum, &c...186
Virchow, Rudolph, Post Mortem Examinations.........................256
Walsh's Physicians' Call-Book and Tablet, and Handy Leger.....761

Biology in England.........................721
Bird Parasites, Eggs of.........................637
Bishops and Doctors......................... 96
Bishop, J. Adams, Treatment of Fractured Jaw without Teeth in the Mouth.........................108
Black Races of Oceanica.........................212
Blasting Without Drilling.........................651
Bliss, D. W., Case of President Garfield.........................601
Blood Enemata.........................699
Bolting Food.........................140
"Bonwill's Method" of Anesthesia.........................136
Boyland, G. H., Case of Strangulated Scrotal Hernia............... 66
Brain Diseases, Some Points in the Treatment of.........................563
Brinton, W., Abortion With Retained Placenta.........................731
Bromhydrate of Quinia in Pernicious Fever. .........................347
Burns, Treatment of .........................195

Page.
Byrd, Harvey, L., Anatomical and Physiological Differences between the Caucasian and African Races.......348
" " Are All the Races of Men Descended from a Single Pair?..........................................274
" " Human Hybridity..........................................101
" " Observations on Yellow Fever and its Treatment..........................................371
" " Preadamite Races of Men..........................1
Cactus Grandiflora and Cimicifuga in Heart Troubles................179
Cancer of the Axilla, Amputation at Shoulder-joint for................686
Cancer of the Breast..........................................703
Capacity for Study in Children..........................................292
Carbuncle, Its Treatment..........................................490
Cardiac Contractions, How to excite when suspended................415
Carr, I. N., Gold *versus* Rubber for Artificial Dentures............233
Case of Strangulated Scrotal Hernia.......................................... 66
Cause of Heat in Mines..........................................210
Cement for Rubber..........................................208
Centripetal and Centrifugal Motions in Animals..........................583
Chaldaic Records at Uxmal..........................................724
Children's Diseases, Clinical Lectures on..........................494
Chisolm, J. J., Spasm of the Intra Ocular Eye Muscles............ 51
Chloral for Toothache..........................................198
Chlorine not an Element..........................................718
Chrysophanic Acid in Skin Diseases.......................... 98
Cleansing the Ear..........................................286
Cocoanut in Tapeworm..........................................416
Cold Air for Domestic Use..........................................358
Collapse in Strangulated Hernia..........................................705
Collodion in Treatment of Sprains..........................................707
Commerce..........................................279
Concentrating Electricity..........................................439
Congenital Atrophy and Digestive Turgescence of the Spleen.....174
Conium in Bronchial Catarrh..........................................327
Conium Maculatum, Physiological Action of.......................... 97
Connecticut Law Against Irregular Practitioners.......................416
Consumption in Cows..........................................433
Cookery for the Sick..........................................342

Page.
Cooking by Electricity......363
Corns, How to remove them......148
Corson, Hiram, The Treatment ot Mammary Abscess......151

### *Correspondence.*

Medical Laws of Alabama...... 75
President American Dental Association...... 75
Reports of Medical Society Proceedings......110
The American Association for the Advancement of Science......654

Croup, Sulphate of Copper in......261
Culture of Children, Aphorisms on......346
Curious Sanitary Discoveries......683
Dental Sound Transmission......676
Dental Therapeusis......550
Dentists' Headaches......419
Destruction of Forests......286
Diaphragm, Absence of......782
Diarrhœa in Typhoid Fever......269
Diet—Raw Oysters......345
Dyspepsia caused by Tight Lacing......340
Diphtheria, Etiology of......273
Diphtheria, Iodine in......119
Diphtheria, Juglans Nigra in......181
Diphtheria, Muriate of Pilocarpine in...... 79
Diphtheria, Salt as a Prophylactic in...... 48
Diseases of Kidneys and Bladder......523
Diseases of the Liver, Spleen and Intestines......443
Death by Drowning......338
Domestic Hygiene......500-565
Dozier, Barton—The proper mode of Vaccinating......105
Death-rate of Rich and Poor......715
Dabney, T. S., Apocynum Cannabinum in Bright's Disease......306
Darnel......640
Earache......699
Early Maternity......274
Earthy Deposit in the Cornea......106
Eczema of the Nipple......697

### *Editorial Department.*

Page.
A City Hospital....177
A Difference in the Count.... 78
A Rhyme Without a Reason....316
A Table of Sounds....113
A Training School for Nurses....595
A Wax Ball in the Vagina Thirty Years....597
Abattoirs....247
About Methods of Medical Teaching....670
Acne Punctata....397
Adams, Dr. A. W.... 39
Adieu....533
Alabama Dental Association....253
Alabama Laws Regulating the Practice of Medicine.... 81
Alexis St. Martin....473
Alumni Associations....120
American Medical Association....319
American Public Health Association....674
Ammonia and Strychnine in Pneumonia.... 38
An American Physician in Germany.... 82
Announcement....533
Annual Meeting of American Dental Association....481
Apocynum Cannabinum in Dropsy....119
Artificial Teeth.... 39
At Our New Home....313
Baltimore College of Dental Surgery....120
Baltimore's Water Festival....534
Bartholow's Cartwright Lectures.... 83
Bellevue Medical College....176
Biology and Johns Hopkins University....247
Birth and Death Rate in Large Cities....477
Bonwill's Method of Rapid Breathing....475
Bromine as a Sedative and Hypnotic....532
Buffalo Lithia Water....184
Business Management of the *Independent Practitioner*....321
Code of Ethics....315, 320
Correspondence....246
Craniotomy and its Alternatives....314

Page.
Cremation....115
Cremation of Dr. Colletti....396
Dartmouth College....760
Death of Prof. Richard O. Cowling....183
Delegates to Medical Convention in London....480
Dental Fees....184
Dental Journalism....122
Diagnosis of Gastric Cancer....478
Diaphragm, Absence of....782
Diphtheria, Muriate of Pilocarpin in....79
Diploma Mills....245
Dr. Bliss' Report of President Garfield's Case....594
Dr. E. F. Cordell....248
Dr. E. Miles Willett....674
Dr. Fred. R. Sturgis....674
Dr. J. Marion Sims....248
Dr. L. P. Yandell....83
Dr. O. W. Wight....760
Dr. Richard Gundry....674
Dr. Theophilus Parvin....674
Drowning of Prof. E. Lloyd Howard....534
Editorial Staff of the *Independent Practitioner*....594
Etiquette....478
For a Mad Dog's Bite....121
Foreign Subscribers....39
Free Baths for Baltimore....391
Goodyear Dental Vulcanite Co....121
Gratuitous Dental Service in the French Primary Schools....760
Grindelia Robusta in Asthma....316
Gunshot Wounds....112
Heart Troubles and Cactus Grandiflora and Cimicifuga....179
Hygiene....315
Hypnotism, Mesmerism, or Syggnosticism....175
Interesting Lawsuit Postponed....532
International Medical Congress....592
Iodine in Diptheria....119
John H. Griscom....395
Leprosy....116

Page.
Life and Mind on the Basis of Modern Medicine........................ 36
Liquor Acidi Phosphorici..............................................480
Malpractice Suit......................................................395
Maternite Hospital....................................................245
Medical Association of Georgia........................................253
Medical and Chirurgical Faculty of Maryland...........................183
Medical Education.....................................................249
Medical Gymnastics for the Phthisical.................................242
Medical Journal Association...........................................319
Meetings of Dental Societies in July..................................397
Meetings of Societies in October......................................598
Metric System......................................................... 76
Mistletoe as an Anti-Hemorrhagic......................................394
Much Craniotomy.......................................................176
National Dental Association...........................................484
New Medical Building for Baltimore....................................392
New Medical College in Philadelphia...................................183
Night Medical Service................................................. 33
Night Suit for Physicians.............................................179
Nostrums and the Clergy............................................... 33
Occupations Suitable for Women........................................246
Odor Mortis...........................................................475
Original Communications............................................... 35
Ourselves.............................................................759
Petroleum in Phthisis................................................. 82
Planetary Parahelia of 1881........................................... 78
Popular Science Department.........................................83, 243
President Garfield....................................................530
Provost of the University of Pennsylvania............................. 77
Public Health Association............................................. 39
Publication of Medical Society Proceedings............................241
Quack Advertisements and the Religious Press..........................318
Quebracho.............................................................317
Quinine...............................................................244
Quinine in Tetanus....................................................393
Quinine in Whooping Cough.............................................182
Salicine..............................................................317
Seats for Shop Girls..................................................474

Page.
Snowden's Binaural Stethoscope........................................ 79
Southwestern Dental Society...........................................597
Special Notice........................................................313
Summer, Malaria, &c...................................................477
Sunnyside.............................................................397
The Academy of Medicine...............................................595
The Am. Gynecological Society.........................................597
The American Medical Association.......................................251
The Baltimore Medical College.........................................594
The Decimal Arrangement...............................................178
The Difference in the Count...........................................182
The Figures 1881...................................................... 78
The Food Adulteration Law.............................................480
The General and Special Education of Dentists.........................758
The International Medical Congress....................................396
The *Independent Practitioner* in New York............................533
The Kidd-Quain Imbroglio..............................................249
The National Dental Association of the United States of America.535
The New York Street Cleaning Bill.....................................396
The Physiological Antagonism of Remedies..............................117
The Woman's Hospital..................................................479
To Advertisers........................................................ 34
To A New Subscriber................................................... 80
Treatment of Yellow Fever.............................................316
Trichinosis...........................................................317
Trichinosis Cured..................................................... 83
Troubles of Journalism................................................116
University of Maryland Medical School.............................120–318
Vaccination...........................................................113
Valedictory...........................................................533
Water and Sand Bags...................................................474
Weekly, Bi-Weekly and Monthly Medical Journals........................ 37
"Who Shall Decide When Doctors," &c...................................316
Water and Sand Bags...................................................474
Education of Dentists.................................................333
Electric Light Notes..................................................435
Electric Light on the Eye, Effects of.................................339
Electric Railway in Berlin............................................356

Page.
Erichsen, H., Quinia in Antiseptic Treatment....239
Ether....225
" Deaths from....783
Ethylate of Soda as a Caustic....782
Exophthalmic Goitre, Electricity in....497
Experiment in Preventive Medicine....784
Expert Testimony....202
Explosion of Dust....651
Explosive Medical Compounds....332
Extirpation of Uterus and Ovaries....268
Extra-Genital Chancre....750
Extracting Teeth During Pregnancy....200
Faye's Theory of the Earth's Crust....217
Feminine Wisdom....140
Fevers, Yellow and Break-bone....9
Filth and Semi-filth Diseases....407
Filth of the Middle Ages....422
Fire from Rubber Goods....579
Fireless Locomotives....437
Food Adulteration Law....508
Foot-prints in the Rock....203
Fractured Jaw, Treatment of....108
Fracture of the Neck of the Femur....754
Galvanism in Hemorrhoids and Habitual Constipation....634
Gastric Cancer, Diagnosis of....478
Garfield, Dr. Bliss' report of Case of President....601
Garrettson, J. E., Surgical Clinic....27
Gelatine, Medicated, in Treatment of Nasal Catarrh....410
German View of the Qualifications of a Physician....196
Getting Above One's Business....198
Giant Powder, Effect of Cold on....575
Glacial Periods....361
Goitre Cured by Fluoric Acid....370
Gold-Mining in Georgia....642
Gold *versus* Rubber for Dentures....233
Gonorrhœal Conjunctivitis, Treatment of....783
Grafting the Tomato on the Potato....362
Grindelia Robusta in Asthma....316

Page.

Griswold, Roger Marvin, A Plea for the More Frequent Use of the Obstetric Forceps....454

" " Some Observations Upon Hereditary Tendencies and Transmissions ...515–587

" Rufus W., Some Observations on the Use of the Lancet.... 60

Growth of Coral....212

Hair Dye with one Preparation....147

Heart-Sounds, Dr. Porcher's Table of....113

Hereditary Tendencies and Transmissions....515-587

Hernia, Case of Strangulated Scrotal.... 66

Hernia, Immediate Cure of....690

High Buildings and Elevators in New York....651

Hippocratic Oath....702

Hoang-Nan Once More....554

Hoarseness, Borax in.... 97

Homœopathy....586

Hot Ice and Cold Fire....331

Hot-water compresses in Tetanus and Trismus....202

Hot Water for Sweaty Feet....710

House Drainage....288

How Soon After Delivery Should a Woman Get Up?....687

How to Prevent Drowning....719

How to Work the Brain....569

How We Are Poisoned....364

Hybridity, Human....101

Hydrastis, Active Principle of....197

Hydrocele in Children, Treatment of....415

Hygiene....581

Hygiene of Food....426

### *Hymeneal.*

Swope—Perine......46. Noel—Folwell......47. Thomas—Harris ......95. Morgan—Evans......95. Crane—Eckard......442. Gleaves —Keene......442. Workman—Bullock......512. Schwartze—Burkart......512. VanBibber—Lusby......512. Shipley—Young......512. Rambo—Jones......512. Harban—Higgins......730.

Page.
Hypnotism, Catalepsy and the Faculty of speech......................138
Indian Hemp, Medical Uses of........................................899
Infection, How to Avoid..............................................433
Infection, the Quality of............................................431
Injection Brou.......................................................779
Injury to the Eye, A Clinical Lecture on.............................659
Insanity and Uterine Diseases........................................426
Insulation of Electric Light Wires...................................360
International Medical Congress...................................137-592
Intestinal Resection.................................................267
Iodine as a Specific in Croupous Pneumonia...........................141
Iodoform in Chronic Nasal Catarrh....................................266
Jaborandi in Asthma..................................................585
Jacobi, Mary Putnam, Case of Facial and Palatine Paralysis...... 69
Kerosene and Croup...................................................266
Laceration of Perineum, Prevention of................................144
Laceration of the Cervix............................................. 48
Lancet, The Use of the...............................................559
Lancet, On the....................................................... 19
Lancet, Observations on the Use of................................... 60
Lead-Line in the Gums, Nature and Origin of..........................763
LeHardy, J. C., On Yellow and Breakbone Fevers....................... 9
Lepra, Parasite in...................................................437
Leprosy in Key West..................................................555
Leprosy in Louisiana.................................................556
Listerine............................................................557
Lithæmia, Nervous Symptoms of........................................777
Longevity............................................................717
Longevity of Brain-Workers...........................................802
Longevity of Medical Men.............................................140
Lunar Display in Colorado............................................368
Macadamised Streets..................................................429
Magic Cabinet........................................................578
Malaria in Italy.....................................................325
Malarial Disease, Notes on Some Cases of.............................666
Malignant Pustule, Treatment of......................................265
Mammary Abscess, Treatment of........................................151
Mammoth Cave of Kansas...............................................579

Page.
Mann, Edward C., The Importance of the Early Recognition of Mental Disease......299
Medical Education......261
Medical Preceptors and College Professors...... 26
Medical Schools......525
Mental Disease, Importance of its Early Recognition......299
Microscopic Analysis of Water......205
Microscopic Structure of Metals......361
Milk as an Article of Diet......779
Mistletoe as an Anti-hemorrhagic......394
Monroe, W. R., Beloved Physicians......310
" " Medical Preceptors and College Professors...... 26
" " Medical Schools......525
" " Responsibilities of Medical Colleges......461
" " Sacredness of the Medical Profession......238
Morphia in Puerperal Convulsions......711
Mortality and Meteorological Report......50–100–150–224–298
Mother's Mark, A Remarkable......418
Mouthwash For Tobacco Consumers......701
Munificent Bequest......716
Nasal Disease, Aids to Diagnosis in......768
Naso-Cranial Osteitis of Syphilitic Origin......564
National Dental Association......484–535

### *Necrological.*

Byrd, W. N......47. Hardee, B. W......47. Knight, Samuel T...... 95. Davis, Wm. H......95. Joynes, Levin S......95. Habersham, J. C. ......132. Otis, George A......134. Cowling, R. O......222. Walls, J. Wm..... 442. Dowell, G..... 513. Hodge, H. L......513. Skoda, Joseph......514. Blackie, G. S......514. Waugh, John......514. Coy, B. F......586. Crowe, J. E......658. Spiegelberg, O......658. Greene, Wm. W......658. Littre, E......658. Bryk, Dr......658. White, Chas. B......730. Holland, J. G......730. Green, J. M......730. Maddux, T. Clay......802. Bache, B. F......802. Holt, A. C......802. Menninger, John......802. Axson, A. F......802. Horner, Fred...... 802. Pancoast, Jas. L......802. Brinton, J. B......802.

Page.
Necrosis, Operation for....632
Neuroma of the Skin....780
New Gaseous Disinfectant....729
New Method for Withdrawing Fluids from the Middle Ear....145
New Pipe Line....646
New Remedies....330
Night Air....728
Nitro-Glycerine in Bright's Disease, &c....143
Nitro-Glycerine in Migraine, Epilepsy, &c....750
Nitro-Glycerine, Therapeutics of....149
Nitrous Oxide Gas....387-464
Observations on Yellow Fever and its Treatment....371
Obstetrical Experiences....553
Obstetric Forceps, Plea For Their More Frequent Use....454
Odor Mortis....475-498
Ocular Symptoms in Different Diseases....775
Only A Vine-Slip....218
Ophthalmia of the New Born, Prevention of....742
Opium-Eating, is it Injurious?....800
Origin of Some Common Plants....716
Osborne, L. C., On the Lancet.... 19
Out-Door Air and Exercise....213
Ovarian Tumor Treated by Incision and Drainage....424
Oxalate of Cerium in Pertussis....704
Ozone in the Atmosphere....722
Pains and Anodynes....425
Palæolithic Osteology....431
Paralysis, Facial and Palatine.... 69
Parker, S. J., Earthy Deposit on the Cornea....106
Peptonate of Mercury....781
Peritonitis, Case of....402
Petrified Forests....638
Pertussis, Modern Remedies for....776
Philadelphia Hospital of Oral Surgery, Surgical Clinic.... 27
Phosphide of Zinc in Locomotor Ataxia....703
Photographs in Natural Colors....430
Phthisis, Petroleum in.... 82
Physiology of the Visual Purple....294

Page.
Picrate of Ammonium in Malarial Fevers........331
Pipe-Line for Natural Brine........644
Pneumonia, Iodine a specific in........141
Pneumonia, Treatment of........141
Poisons, Influence of Race Upon the Action of........676
Polyuria Caused by Ergot........147
Post Partum Hemorrhage........328
Potentization Giving Way........326
Preadamite Races of Men........ 1
Precocity A Sign of Inferiority........291
Prehistoric Mining in North Carolina. ........2[illegible]6
Proper Treatment of Common Eye Diseases........752
Prostitution, Regulation of, and Repression of Syphilis........573
Punishing Children........641
Pus Dyscrasia........ 23
Puzzle for the Water Scientists........645
Quebracho........317
Quinia From Coal Tar........195
Quinia in Antiseptic Treatment........239
Races of Men; Are All Descended From a Single Pair?........274
Rabies, A Possible Preventive of........799
Raindrops, Hailstones and Snowflakes........796
Reality of Hypnotic Phenomena........724
Recognition, A Long Desired........418
Rectal Douche........146
Rectal Exploration and Diagnosis........686
Removing Nitrate of Silver Stains........147
Resonant Sand........643
Resorcine........439
Responsibilities of Medical Colleges........461
Richmond Crowns........417
Rock-Boring Ephemera ........217
Roosa, D. B. St. John, A Clinical Lecture on Ophthalmology—
Injury to the Eye........659
Ruff, W. H., Adam a Nation........171
Rupture, Radical Cure of........370
Sacredness of the Medical Profession........238
Sad End of a Promising Life ........137

Page.
Salicine....317
Salicylic Acid for Cold in the Head....703
Sanitary Insurance....288
Sanitation and Christianity....283
Self-Mutilation, Rare Case of....714
Separating Gold, Silver and Copper Alloy by Electrolysis....290
Sewage in Oysters....801
Sewering New Orleans....412
Shurley, E. L., On the Treatment of Diseases of the Liver, Spleen and Intestines....443
" " Treatment of Diseases of the Kidneys and Bladder....523
Sims, Dr. J. Marion....99
Sleeplessness From Thought....280
Small-Pox and Anti-Vaccination....801
Smoke and Fog of London....421
Small-Pox in a Fœtus....707
Spasm of the Eye-Muscles....51
Spray, Fate of the....412
Stammering and its Cure....726
Stertorous Breathing in Apoplexy....772
Storm Water and House Drainage....339
Successful Doctor (Poem)....30
Sudden Death by Chloroform....712
Sugar from Rags....570
Suicide by Dynamite....774
Sulphurous Acid as a Bleach....649
Surgico-Anatomical Study of the Gunshot Wound of President Garfield....627
Suture of the Radial Nerve....705
Sweating of Consumptives, Import of....413
Syphilis, Contribution to the Study of Inherited....744
Syphilis in Different Countries....780
Syphilitic Alterations of the Teeth....708
Syphilitic Tumors of Bone in Infancy....710
Telegraph Cable in Sewers....441
Telegraphing Without Wires....221
Temperature of Sleeping Rooms....340

Page.
Testing Seeds....639
That Bedroom....434
The Epizootic Disease.... 47
The Moquis....292
The Photophone....209
The Sunflower....370
Therapeutic Pearls at Random Strung....
43–90–128–190–259–323–400–488–541–652
Tinctura Antacrida....414
Tobacco—A Parable....344
Tonga....782
Tonsillotomy and Virility....559
Toothache, Suggestions in....412
Tooth Pulp, Digestion of....201
Tracheotomy Without Tubes....197
Trance from Fright....329
Transmission of Power by Electricity....643
Treatment of Miner's Disease....369
Treatment of Burns....341
Treatment of Diseases of the Kidneys and Bladder .... 523
Trichinæ in Man....284
Trichinosis....317
Trigeminus, section of.... 706
Turnbull, Laurence, Anæsthetics and Narcotics....161
" " Ether....225
" " Nitrous Oxide Gas....387–464
Typhoid, the Poison of....707
Underclothing, Frequent Changes of....282
Urethritis, Is There a Specific?....636
Uterine Displacements, Curability of....781
Uterus, Removal of, for Cancer....771
Uterus, Rupture of, Treated by Drainage....684
Vaccinated and Re-vaccinated....560
Vaccinating, Proper Mode of....105
Vaccination Experiences....577
Value of Mental Tension....718
Valuable Invention....576
Vital Statistics, Curiosities of.... 96

Page.
Weight of the brain and its functional ability .................................293
Wentworth, T. J., The Successful Doctor.................................... 30
Weisse, F. D., Surgico-Anatomical Study of the Wound of President Garfield. .............................................................627
What Air Shall We Breathe at Night...........................................357
What Volcanoes Are Not .................................................723
Wheat, Introduction of, into America.............................................416
Whooping Cough, Quinine in................................................. 182
Why Does Labor Come on.................................................778
Woman's Hospital, The.................................................493
Yellow Fever and its Treatment................................. .371 544

TO ADVERTISERS.—This journal offers unusual advantages as an advertising medium. Its circulation is large and constantly increasing among the best members of the profession, in almost every city, town and hamlet in the United States; and it goes already into Canada, the West Indies, Mexico, and nearly every one of the South American States. Its circulation is gradually extending in Europe, and a few copies go to other parts of the eastern continent. To all those, therefore, who have valuable inventions, implements, new preparations of medicine, mineral waters, Summer and Winter resorts for invalids, etc., which they wish to make favorably known to the profession, its advertising department offers unusual advantages and facilities. All reputable medical colleges—none others need apply—would find it likewise to their interest by calling the attention of students to them through its advertising department. It was decided in the inseption of the enterprize to admit no advertisement into the journal that was not *first-class* in *all respects*, and the same rule will continue to govern in the future as the past in that regard. From all, therefore, who would have a *quid pro quo* for what they offer to the public we invite advertisements! The rates and terms will be in accordance with what is customary with the best journals of this country. We very much prefer quality to quantity in our pages, and we promise our readers and patrons that there shall always be more professional matter than advertisements in the respective issues of the journal.

Foreign Subscribers.—In order to meet the extra postage, etc., we find it necessary to raise our subscription rates in all foreign countries except Canada, to $2.50 per annum, in advance. Messrs. C. Ash & Sons, Broad Street, Golden Square, London, W., or at their branches in Liverpool, Manchester, Paris, Berlin, Vienna, Hamburgh, Copenhagen, and St. Petersburgh, and Dr. Sam. D. Rambo, of Rio De Janeiro, 130 Ruado do Duridor, Brazil, will receive orders and money for the Independent Practitioner.

### HYMENEAL.

"Domestic happiness, thou only bliss
Of Paradise that has survived the fall."

SWOPE—PERINE.—Married, November 11th, Benedict W. Swope, Esq., to Miss Evalyne, daughter of Dr. George H. Perine of

116 West 42nd Street, New York. We regret our inability to be present, but extend to the nuptial pair our congratulations and sincere wishes for their continued happiness.

NOEL—FOLWELL.—Dr. Llewellyn G. Noel, of Nashville, Tennessee, to Nannie Folwell, of Memphis, Tennessee, Calvary Church, December 2nd.

"Oh! if there be an elysium on earth"—

### THE EPIZOOTIC DISEASE.

Beginning a month ago, in the extreme north of New England, the so-called "epizootic disease" has extended southward, until now

WILL *YOU* PLEASE SEND IN *YOUR* SUBSCRIPTION MONEY *NOW?*

### CONTENTS FOR JANUARY, 1881.

ORIGINAL COMMUNICATIONS.

Night Medical Service .......... 33

Nostrums and the Clergy .......... 33

To Advertisers .......... 34

Original Communications and other Contributions 35

Pure and Reliable Medicines .......... 36

Life and Mind on the Basis of Modern Medicine. 36

Weekly, Bi-Weekly and Monthly Journals ...... 37

The Use of Ammonia and Strychnine in the Treatment of Pneumonia .......... 38

This Number of the Journal .......... 38

The Annual Meeting of the American Public Health Association .......... 39

Dr. A. Wellington Adams .......... 39

Foreign Subscribers .......... 39

Artificial Teeth .......... 39

### *U. S. POSTAL LAW EXTRACTS.*

—:o:—

Postmasters are responsible to publishers for the payment of all papers, journals, magazines, etc., not delivered to the persons to whom they are addressed, unless legally accounted for.

Any person who takes a paper from the post-office, whether directed to his name or another, or whether he has subscribed or not, is responsible for the pay.

WE publish the above extracts, not with the intention of forcing our journal upon anyone. We do not send it out as an advertising sheet, as we have no goods to sell. It is purely an Independent Monthly, supported by its subscribers and advertisers. We, therefore, courteously request all who may receive a copy, to notify us if it is not acceptable, and they do not wish to pay for it. We have to resort to this "method" as we have no agents. However, we ask no one to subscribe who is not pleased and satisfied that it is worth the subscription price. Each Number will contain fifty pages of reading matter, making an Annual Volume of six hundred pages for $2.00.

This column will be sold for advertising space six month during the year at *special rates.*

Money is at OUR RISK ONLY when sent by Post Office Money Order, Registered Letter, or Check on Banks in New York City or Baltimore, made payable to Dr. B. M. Wilkerson, 68 N. Charles street, Baltimore, Md.

If you prefer to send check on your private bank, unless located in the cities of Baltimore or New York, please add 25 cents for collection.

